# Integrating technologies for comprehensive anorectal assessment: steps toward objective functional diagnosis

**DOI:** 10.3389/fmed.2025.1740123

**Published:** 2026-02-04

**Authors:** Hans Gregersen

**Affiliations:** California Medical Innovations Institute, San Diego, CA, United States

**Keywords:** anorectal, assessment, defecatory disorder, Fecobionics, functional

## Introduction

Anorectal functional disorders, particularly dyssynergic defecation (DD), represent a major diagnostic and therapeutic challenge in modern gastroenterology. Despite the availability of multiple investigative tools, including anorectal manometry (ARM), balloon expulsion test (BET), defecography, and electromyography, clinical interpretation often remains ambiguous because each technique assesses only a subset of the biomechanical and neuromuscular events underlying defecation. The recent study by Lamichhane et al. (1) in Gastroenterology, as well as two other recent publications by the same group, provide an important conceptual advance by integrating synchronous pressure and imaging measurements to achieve a multidimensional evaluation of anorectal function. This Opinion article aims to contextualize that work, discuss its implications for the evolution of multimodal testing, and reflect on how new technologies may eventually redefine diagnostic paradigms in functional proctology.

## Multimodal integration in anorectal diagnostics

The diagnosis of a defecatory disorder requires Rome IV symptom-based criteria (2) and demonstration of abnormal evacuation by at least two independent modalities such as ARM, BET, defecography, or electromyography. The rationale for this composite requirement is clear: no single technique provides a comprehensive picture of the complex physiological events involved in coordinated defecation. The paper by Lamichhane et al. (1) addresses this long-standing challenge by targeting different functional aspects. In clinical practice, coordinating and interpreting the results of several separate procedures are cumbersome, capital-expensive, time-consuming, and often inconclusive, especially when results are borderline or discordant. Their paper responds to the first of mentioned unmet clinical needs (1), integrating manometric pressure data with real-time imaging of pelvic floor motion and anorectal angle deformation. Their synchronous approach allows simultaneous capture of structural and functional information, thereby bridging the gap between physiology and anatomy in a single procedure in DD patients, e.g., by showing simultaneous pressure changes and descent of the pelvis floor during evacuation. The combined ARM with defecography parameters provides real-time multidimensional evaluation of complementary modalities—something technically and logistically demanding. The results reveal distinct phenotypic patterns and the authors use cluster (k-means) analysis to identify subgroups that may correspond to different pathophysiological mechanisms.

The idea of integrating technologies is, however, not new (see below). Table 1 shows four evolutionary stages of integration or approaches to testing in clinical work and research. This spans from the serial test approach where one test determines if a subsequent test is done (Stage I). This is a very common test pattern in clinical work. The second stage represents serial testing that already has been decided before doing the first test. This is common in studies where the diagnostic criteria dictates that at least two tests must show abnormality. Stage III represents an approach with simultaneous use of current state-of-the-art tests, such as the study by Lamichhane et al. (1). Since such tests are cumbersome, equipment-demanding and not patient-friendly, they are primarily for research purposes. Stage IV is the ultimate stage where the tests are integrated into a single device for both clinical research purposes. Stage IV tests are already available.

**Table 1:** Evolutionary stages in clinical testing (serial, combined in parallel, and integrated).

**Stage**	**Approach**
Stage 1	Serial tests where the result of a test determines if other tests are done. The broad clinical diagnostic approach.
Stage 2	Serial tests but usually not dependent on previous tests. The typical approach used for diagnostic criteria where at least two tests must show abnormality.
Stage 3	Two current state-of-the-art tests done simultaneously but with different data acquisition systems. Typical for research purposes by combining manometry and imaging.
Stage 4	New integrated technology combining multiple technologies into a single test and with a single data acquisition system. More patient-friendly and objective testing with high tolerability.

## Historical and technological context

The concept of multimodal measurement in gastrointestinal physiology is not new. Ratuapli et al. (3) as well as others suggested to use ARM and imaging technology together. Over the past two decades, several groups have explored simultaneous or sequential pressure-imaging methods to study esophageal (4), stomach (5), intestinal (6), and anorectal mechanosensation (7, 8). These studies laid the groundwork for the future integration, demonstrating that mechanical strain and pressure gradients provide complementary insights into wall biomechanics. Other studies in esophageal testing with the pairing of high-resolution manometry with impedance monitoring revolutionized the assessment of peristaltic function by linking pressure waves to bolus transit.

Lamichhane et al. extend above multimodal principle by achieving physiological data by synchronization between modalities, thereby enabling time-resolved correlation of pressure peaks with dynamic structural changes during evacuation. Nevertheless, MRI-based studies suffer from several inherent limitations. They typically require the patient to adopt a semi-physiological position, often lying on the side or sitting within the scanner, which does not fully replicate natural defecation dynamics. The use of contrast paste or balloon simulants can also alter rectal compliance and sensory perception. Additionally, movement artifacts, synchronization errors, and high operational costs hinder the scalability of such methods. For these reasons, combined ARM-MRI defecography is unlikely to gain widespread clinical use since it is cumbersome and not patient-friendly but can be a useful research tool as demonstrated (1). While scientifically innovative, the practical implementation of synchronous ARM and MRI defecography remains logistically demanding. The setup requires specialized equipment, expert coordination, and prolonged testing time, which together limit its applicability in routine clinical environments. The authors correctly acknowledge these constraints while emphasizing the potential value of their approach as a research tool that deepens mechanistic understanding.

Parallel efforts have pursued more physiological and patient-friendly technologies for the same purpose. Among these, the Fecobionics system represents a promising stage IV development (9–11).

## Emergence of Fecobionics and other integrative technologies

In this context, novel technologies such as Fecobionics have emerged as promising successors to multimodal imaging. Fecobionics is a soft, electronic, stool-mimicking FDA-cleared device that measures pressure, bending angle (a proxy of the anterior anorectal angle during passage), orientation, position, and fecal flow resistance during defecation. The system provides real-time, fully electronic readouts of biomechanical pathophysiological parameters that previously required separate tests. In newer iterations, the device incorporates impedance planimetry (EndoFLIP) to assess cross-sectional geometry, effectively uniting the functions of ARM, BET, and defecography in one test.

Unlike MRI-based assessments, Fecobionics operates under near-physiological conditions, with patients in natural posture performing a familiar task. The resulting data are rich, objective, and amenable to automated analysis. Technological studies on the bench and in patients have validated the technology (12–14). Clinical studies have confirmed that Fecobionics can differentiate between DD phenotypes and other constipation subtypes, offering actionable insights without the discomfort associated with invasive or radiological procedures (9–11). Therefore, the ARM–MRI integration proposed by Lamichhane et al. may be seen as a transitional step between conventional multimodal workflows and the emerging generation of self-contained diagnostic systems.

## Data-driven stratification and the role of clustering analysis

One of the most innovative aspects of the Lamichhane et al. (1) study is the application of k-means clustering to categorize DD patients into subgroups based on combined physiological and structural parameters. Such data-driven methods are increasingly used in gastroenterology to explore heterogeneity within complex disorders. However, the approach requires careful interpretation. Clustering algorithms are sensitive to the choice of variables, their normalization, and the predetermined number of clusters. Without external validation, the resulting phenotypes may remain descriptive rather than clinically actionable. Future work should include reproducibility studies, cross-validation across cohorts, and correlation of cluster membership with treatment outcomes. The question remains whether similar conclusions could be reached more simply. For clinical practice, reproducible thresholds or algorithmic scoring systems may ultimately prove more practical.

Beyond unsupervised learning tools such as k-means clustering, emerging supervised machine learning techniques hold promise for translating physiological data from Stage IV tests into predictive diagnostic models. Integrating multimodal data streams from stage IV devices could enable automated classification of DD types, prediction of biofeedback therapy response, and objective measurement of treatment efficacy. Such data-driven frameworks would move the field toward personalized diagnostics, where technology informs individualized management strategies.

## Standardization and future directions

Despite these advances, the field continues to face significant barriers to standardization. Multimodal tests generate large and complex datasets that lack universally accepted interpretive criteria. Establishing normative reference databases is essential to ensure inter-center comparability and to define pathophysiological thresholds. The diagnostic criteria, which currently require abnormal findings in two separate modalities, may eventually be revised to accommodate integrated stage IV devices capable of collecting multiple data types simultaneously in a single test. At that point, diagnostic definitions could shift from test-based to function-based frameworks.

Patient comfort and test accessibility should remain central priorities. Many patients with functional bowel disorders are hesitant to undergo invasive or embarrassing procedures, and reducing discomfort can significantly improve compliance and diagnostic yield. Furthermore, machine learning has potential to move beyond unsupervised clustering toward predictive analytics and personalized management. Lastly, future research should examine how these new measurements align with symptom severity and quality-of-life indices, bridging the gap between metrics. Portable, automated, and physiologically representative Stage IV tools will likely dominate future clinical workflows. Parallel to technological innovation, collaboration across physiology, engineering, and data science disciplines will be essential to translate these developments into clinically meaningful standards.

## Discussion

Despite not presenting a new idea, the study by Lamichhane et al. (1) represents an important proof of concept demonstrating that simultaneous measurement of pressure and structural changes is feasible and informative. Their results underscore the potential of multimodal integration to refine diagnostic precision, characterize pathophysiological heterogeneity, and lay the foundation for data-driven classification of anorectal disorders. However, the practical challenges of MRI-based approaches underscore the need for simplification, automation, and patient-centered design in future iterations. Such Stage IV tests have already been validated and provide data that are easier to analyze and with better correlation to symptoms of the patients.

As the field progresses, the vision of a single, comprehensive test that objectively captures the mechanical, sensory, and neural dimensions of defecation appears increasingly attainable. Systems like Fecobionics exemplify this trajectory, providing high-fidelity physiological data while maintaining patient comfort and procedural efficiency. The combination of advanced sensing technology with modern analytical tools such as machine learning and supervised tools promises to transform the current subjective and fragmented diagnostic paradigm into one that is quantitative, standardized, and predictive.

Ultimately, technological progress must be matched by parallel evolution in clinical interpretation and patient engagement. The integration of multidimensional data is only meaningful if it informs better clinical management decisions and outcomes. Studies like that of Lamichhane et al. catalyze this transformation by highlighting both the possibilities and limitations of current multimodal strategies. As innovation continues, gastroenterology moves closer to achieving comprehensive, objective, and patient-friendly diagnosis of functional anorectal disorders. In conclusion, assessment of defecatory function is developing rapidly with recognition of the need for combined testing of modalities. In the author's opinion, the ultimate goal is to shift diagnostic definitions from test-based to function-based frameworks.
